# FK228 suppress the growth of human malignant pleural mesothelioma tumor independent to epithelioid or non-epithelioid histology

**DOI:** 10.1186/s10020-024-00835-6

**Published:** 2024-05-31

**Authors:** James Mei-Lin Chan, Yuan-Ching Chang, Hua-Chen Chan, Hsiu-Chuan Chan, Wei-Chin Chang, Liu-Fang Wang, Tung-Hu Tsai, Yu-Jen Chen, Wen-Chien Huang

**Affiliations:** 1 Department of Surgery, Mackay Memorial Hospital, Taipei, Taiwan; 2 Institute of Traditional Medicine, School of Medicine, National Yang Ming Chiao Tung University, Taipei, Taiwan; 3 Department of Medicine, Mackay Medical College, New Taipei City, Taiwan; 4 Department of Medical Research, Mackay Memorial Hospital, New Taipei City, Taiwan; 5 Department of Medical Laboratory Science, College of Medicine, I-Shou University, Kaohsiung, Taiwan; 6 Center for Lipid Biosciences, Department of Medical Research, Kaohsiung Medical University Hospital, Kaohsiung, Taiwan; 7 PhD Program in Life Sciences, College of Life Science, Kaohsiung Medical University, Kaohsiung, Taiwan; 8 Pathology Department, Taipei Medical University Hospital, Taipei, Taiwan; 9 Department of Radiation Oncology, MacKay Memorial Hospital, Taipei, Taiwan

**Keywords:** Human malignant pleural mesothelioma (hMPM), Epithelioid, Sarcomatoid, Non-epithelioid, Romidepsin (FK228)

## Abstract

Human malignant pleural mesothelioma (hMPM) is an aggressive, rare disease with a poor prognosis. Histologically, MPM is categorized into epithelioid, biphasic, and sarcomatoid subtypes, with the epithelioid subtype generally displaying a better response to treatment. Conversely, effective therapies for the non-epithelioid subtypes are limited. This study aimed to investigate the potential role of FK228, a histone deacetylase inhibitor, in the suppression of hMPM tumor growth. We conducted a comprehensive analysis of the histological and molecular characteristics of two MPM cell lines, CRL-5820 (epithelioid) and CRL-5946 (non-epithelioid). CRL-5946 cells and non-epithelioid patient-derived xenografted mice exhibited heightened growth rates compared to those with epithelioid MPM. Both CRL-5946 cells and non-epithelioid mice displayed a poor response to cisplatin. However, FK228 markedly inhibited the growth of both epithelioid and non-epithelioid tumor cells in vitro and in vivo. Cell cycle analysis revealed FK228-induced G1/S and mitotic arrest in MPM cells. Caspase inhibitor experiments demonstrated that FK228-triggered apoptosis occurred via a caspase-dependent pathway in CRL-5946 but not in CRL-5820 cells. Additionally, a cytokine array analysis showed that FK228 reduced the release of growth factors, including platelet-derived and vascular endothelial growth factors, specifically in CRL-5946 cells. These results indicate that FK228 exhibits therapeutic potential in MPM by inducing cytotoxicity and modulating the tumor microenvironment, potentially benefiting both epithelioid and non-epithelioid subtypes.

## Introduction

Malignant pleural mesothelioma (MPM) is a rare and aggressive tumor with a poor prognosis. The World Health Organization (WHO) classification recognizes three major subtypes: epithelioid, biphasic, and sarcomatoid (Sauter et al. 2022). The epithelioid subtype is the most common, accounting for 60–80% of cases, while the sarcomatoid subtype represents 10% of cases, with the biphasic MPM accounting for the remaining 30% (Husain et al. 2018; Zandwijk et al. 2013). Epithelioid MPM is associated with a more favorable prognosis, whereas the sarcomatoid subtype exhibits worse outcomes with lower response rates to therapy (Meyerhoff et al. 2015).

Standard therapy for MPM typically involves a combination of cisplatin and pemetrexed or cisplatin alone (Zandwijk et al. 2013; Meerbeeck et al. 2005). However, MPM is characterized by inherent chemoresistance, particularly the sarcomatoid subtype, which leads to low response rates (Mansfield et al. 2014). Epithelial-mesenchymal transition (EMT) processes have been linked to cisplatin resistance, with genes associated with EMT found to be enriched in the sarcomatoid subtype, suggesting the potential involvement of EMT in cisplatin resistance (Reynies et al. 2014). Dysregulation of cell cycle control has also been observed in MPM, with the loss of negative regulators, such as cyclin-dependent kinase inhibitors, and overexpression of cyclin-dependent kinases (Crispi et al. 2010). Comparative analysis of normal pleural tissue and MPM patient samples demonstrated the overexpression of genes involved in cell cycle checkpoints, including CDK1/2 (cyclin-dependent kinase 1) and CDKN2C (cyclin-dependent kinase 2 C, p18/INK4C) (Romagnoli et al. 2009). Targeting the cell cycle has emerged as a promising therapeutic strategy for MPM (Romagnoli et al. 2009; Fassl et al. 2022). Consequently, we hypothesized that downregulation of the cell cycle could potentially serve as a therapeutic approach for sarcomatoid mesothelioma.

Histone deacetylases (HDACs) play a crucial role as epigenetic regulators, modulating histone tail modifications, chromatin structure, and gene transcription. Additionally, they are involved in post-translational modifications and the regulation of protein acetylation, which are implicated in cancer progression (Li and Seto 2016; Yu et al. 2002). Increased HDAC expression has been observed in malignant cells and contributes to the acquisition of malignant phenotypes during cancerogenesis (Yoon and Eom 2016). HDACs are classified into four classes: class I (HDACs 1, 2, 3, and 8), class II (HDACs 4, 5, 6, 7, 9, and 10), class IV (HDAC 11), and class III (sirtuin family: SIRT1-SIRT7) (Holbert and Marmorstein 2005). Class I HDACs stimulate cell proliferation and inhibit apoptosis and are often overexpressed in gastrointestinal, prostate, and breast cancers (Choi et al. 2001; Halkidou et al. 2004; Zhang et al. 2005). Moreover, HDACs play critical roles in cell cycle regulation. HDAC1, for instance, influences cell cycle progression by modulating the activation of p21-mediated cyclin-CDK complexes, E2F, and p53 deacetylation (Zupkovitz et al. 2010; Martinez-Balbas et al. 2000; Ito et al. 2002). Consequently, inhibition of HDAC activity or targeting cell cycle checkpoints have emerged as potential therapeutic strategies (Romagnoli et al. 2009; Krug et al. 2015). Despite these advances, the development of effective therapies for refractory sarcomatoid mesothelioma remains a critical clinical challenge.

The process of programmed cell death, or apoptosis, is comprised of distinct morphological characteristics and energy-dependent biochemical mechanisms. For many years, apoptosis has been the main goal of clinical oncology in the development of effective therapies to eliminate cancer cells. Two main mechanisms of apoptosis depend on whether caspases are involved in “caspase-dependent” and “caspase-independent” apoptosis. Caspase-dependent apoptosis includes both the intrinsic and extrinsic pathways. The intrinsic pathway begin within a cell and is mitochondrial-mediated, where apoptosis is initiated by DNA damage, heat shock, and oxidative stress. In contrast, the extrinsic pathway starts outside the cell and is receptor-mediated and initiated by receptor ligation, trimerization, and recruitment of adapter molecules, such as FADD and TRADD, to the death domain (DD), which forms a complex known as the death-inducing signaling complex (DISC). Both the intrinsic and extrinsic pathways initiate highly sophisticated and complex energy-dependent cascades of caspase-involving molecular events that converge on the same terminal or execution pathway. This pathway, initiated by the cleavage of caspase-3, leads to DNA fragmentation, nuclear protein degradation, cytoskeletal changes, and ligand expression for phagocytic cell receptors, resulting in uptake by phagocytic cells (Carneiro and El-Deiry 2020; Elmore 2007). Caspase independence is mainly regulated by mitochondria and can be induced by intracellular molecules, natural compounds, synthetic molecules, and repurposed drugs. In response to the stimulus, the mitochondria release cleaved apoptosis-inducing factor (AIF) and Smac/DIABLO, which then induce chromatin condensation and DNA fragmentation (Bhadra 2022).

The tumor microenvironment (TME) consists of different cell types and several molecules secreted by tumor cells, stromal cells, immune cells, and the extracellular matrix (ECM). The TME plays a pivotal role in tumor differentiation, epigenetic modulation, dissemination, and immune evasion (Labani-Motlagh et al. 2020).

Tumor cells release cytokines and non-coding RNA, initiating a dynamic and reciprocal relationship with the surrounding tissue, resulting in a complex and continually evolving entity. The composition of the TME varies among different tumor types and actively promotes cancer progression. In response to a hypoxic and acidic microenvironment, the TME orchestrates angiogenesis to restore the oxygen and nutrient supply while eliminating metabolic waste. This angiogenic process is driven by pro-angiogenic factors, such as VEGF, bFGF, and PDGF, which are secreted by tumor cells or immune cells within the TME (Anderson and Simon 2020; Jiang et al. 2020).

Romidepsin (FK228), a natural product derived from *Chromobacterium violaceum* is a potent inhibitor of class I HDACs, exhibiting substantial anticancer activity. Recent studies have reported synergistic effects when combined with dual HDAC/PI3K inhibition, leading to apoptosis induction in human cancer cells (Pojani and Barlocco 2021; Yan et al. 2019). HDAC inhibitors also modulate the tumor microenvironment, providing additional anticancer benefits (Li and Seto 2016). In this study, we investigated the effects of FK228 on the inhibition of tumor cell proliferation and alteration of cytokine expression in refractory sarcomatoid mesothelioma. By exploring these aspects, we hope to contribute to the understanding of FK228’s potential as a therapeutic strategy for sarcomatoid mesothelioma and address the critical clinical challenge of refractory diseases.

## Materials and methods

### Patient-derived xenograft (PDX) xenografting MPM models

Patient-Derived Xenografts (PDXs) were established from human malignant pleural mesothelioma samples, previously characterized by our research team (Wu et al. 2017). Two PDX models, namely #Meso-17 (sarcomatoid subtype) and #Meso-24 (epithelioid subtype), were kindly provided by Prof. Marc de Perrot of Toronto General Hospital, Toronto, Canada, and were randomly selected for the study. PDXs were implanted subcutaneously into the right flank of 6-week-old male NOD/SCID mice. Three weeks after implantation, when the subcutaneous tumors reached a diameter of 4–5 mm, the mice were divided into three groups: saline, cisplatin, and FK228. Cisplatin (3 mg/kg) and FK228 (2 mg/kg) were administered via intraperitoneal injection with a 29-gauge needle on Days 0, 7, 14, and 21, respectively. Tumor size was monitored weekly from Day 0 onwards. Tumor dimensions were measured using a microcaliper, and the tumor volume was calculated using the formula V = πab²/6 (where a represents the longest length and b represents the perpendicular width). The mice were euthanized according to the institutional ethics protocol when the tumor volume reached 1500 mm³ or when signs of ulceration were observed. All animal experiments were performed according to a protocol approved by the Institutional Ethical Board of Mackay Memorial Hospital (No. MMH-A-S 110 − 28).

### Histological analysis

*Human mesothelioma cell culture.* Two human mesothelioma cell lines, NCI-H28 [H28] (ATCC® CRL-5820™) and NCI-H2452 [H2452] (ATCC® CRL-5946™), were obtained from ATCC (American Type Culture Collection). The cells were cultured in RPMI-1640 medium supplemented with 10% fetal bovine serum (FBS) and 1% penicillin-streptomycin. The cultures were maintained in a humidified atmosphere containing 5% CO2 at 37 °C. Cells were seeded on chamber slides and subjected to Liu staining (Li-Tzung Biotechnology Inc.) for further analysis.

*Tumor tissue collection and processing.* Formalin-fixed paraffin-embedded blocks of PDX tissue from euthanized NOD/SCID mice were sectioned at a depth of 4 μm and stained with hematoxylin and eosin (H&E). Images were captured using an Optical Microscope Imaging System (Zeiss) and analyzed using ImageJ software (NIH).

### Cytotoxicity analysis of mesothelioma cell lines

A short-term cytotoxicity analysis was conducted to assess the impact of cisplatin and FK228 on various human mesothelioma cell lines by quantifying the number of viable cells. Human mesothelioma cells (CRL-5820 and CRL-5946) were seeded into 96-well flat-bottom culture plates (Corning Life Sciences) at a density of 2500 cells per well. The cells were then treated with either PBS or FK228 (MedChemExpress) at different concentrations: 0.1, 1, 5, and 10 nM. After an additional 3 days of incubation, the number of viable cells was determined using the MTT (3- (4,5-dimethylthiazol-2-yl)2,5-diphenyl tetrazolium bromide) cleavage assay. Absorbance at 540 nm was measured using an ELISA reader to quantify the number of viable cells. To confirm caspase-dependent apoptosis, a general caspase inhibitor, z-VAD-fmk (z-VAD), was used.

A long-term cytotoxicity analysis was conducted to evaluate the effects of the treatments on CRL-5820 and CRL-5946 cells. Initially, the cells were seeded in 6-well plates at a density of 2000 cells per well. Subsequently, the cells were exposed to different treatments: DMSO (control), 2 µg/mL cisplatin, or FK228 at concentrations of 0.25, 0.5, 1, 2, and 4 nM. After 24 h, the medium was replaced with a new culture medium. Following a 14-day incubation period, the culture medium was removed and the cells were washed twice with cold PBS. The plates were then stained with methylene blue, and the number of positive colonies, each containing more than 50 cells, was counted to assess the long-term cytotoxic effects of the treatments.

### Cell cycle analysis

Human CRL-5820 and CRL-5946 mesothelioma cells were seeded and cultured in 6-well plates at a density of 1 × 10^5^ cells per well for 24 h and then treated with or without DMSO or 5 nM FK228 for 1, 2, and 3 days. Cells were harvested, washed with PBS, and fixed in 70% ethanol at 4 °C overnight. Subsequently, the cells were incubated with 1% RNase A at 37 °C for 30 min and with propidium iodide solution (ThermoFisher Scientific) at 4 °C for 30 min in the dark. The DNA content of the cells was measured using a FACSCalibur flow cytometer (BD Biosciences). The experiment was independently repeated six times, and the data were analyzed using MultiCycle DNA content and cell cycle analysis software (Kaluza Analysis Software version 2.1, Beckman Coulter).

### Assessment of apoptosis by TUNEL assay

Apoptosis was verified using a TUNEL assay. Briefly, both CRL-5820 and CRL-5946 cells were exposed to FK228 (5 nM) with or without z-VAD (200 µM) at 37 °C for 72 h. Subsequently, cells were collected, washed with PBS buffer, and fixed with 4% paraformaldehyde for 20 min at room temperature. After washing three times with PBS buffer, the fixed cells underwent the TUNEL assay using the APO-BrdU™ TUNEL assay kit (ThermoFisher Scientific) following the manufacturer’s protocols. Nucleus counterstaining was performed by incubating the cells with propidium iodide at room temperature for 10 min. The fluorescent-labeled cells were analyzed using the FACSCalibur flow cytometer (BD Biosciences) and software (Kaluza Analysis Software version 2.1, Beckman Coulter) was used to quantify apoptotic cells.

### Western blot

Cells were homogenized in radioimmunoprecipitation assay (RIPA) extraction buffer (Sigma-Aldrich). Cell lysates were separated by sodium dodecyl sulfate–polyacrylamide gel electrophoresis and subsequently transferred to a polyvinylidene difluoride membrane. To detect specific proteins, membranes were probed with primary antibodies against vimentin (Cell Signaling Technology) and a cell cycle checkpoint antibody kit (Cell Signaling Technology). The signals were visualized and recorded using a G: Box iChemi XT image analyzer (Syngene).

### Cytokine array

Cytokine production and quantification in CRL-5820 and CRL-5946 cells were assessed using a human cytokine array panel. Cells were treated with either DMSO (control) or FK228 (5 nM) at 37 °C for 72 h. The cell culture supernatant was collected and subjected to cytokine analysis using a Proteome Profiler Human Cytokine Array Kit (R&D Systems). Three replicates per sample and two replicates per cytokine were performed. This array panel allowed the profiling of 105 different cytokines. To perform the assay, membranes were blocked with 1X assay diluent, followed by incubation overnight with cell culture supernatant at 4 °C. Subsequently, the membranes were incubated with biotin-labeled anti-cytokines, followed by incubation with avidin-horseradish peroxidase. Membranes were developed using enhanced chemiluminescence (Thermo Fisher Scientific), and signals were detected using a G: Box iChemi XT image analyzer (Syngene). Quantification of the cytokine spots (dots) on the membranes was performed using the ImageJ software (version 1.45; NIH). The integrated signal density for each cytokine was determined and normalized against internal standards provided by the array. Fold differences in cytokine secretion were reported between the treatment and control groups, providing insights into the effect of FK228 on cytokine production.

### Statistical analysis

All data were presented as relative frequencies for discrete responses and as the mean ± standard deviation for continuous responses and were processed by Graphpad Prism 9, Kaluza Analysis Software version 2.1, and Backman Coulter. A Student’s t-test was used to compare differences between two groups. *P* < 0.05 was considered statistically significant. GraphPad Prism 9 (GraphPad Software, San Diego, CA, USA) was used to perform all statistical analyses. Statistical significance was set at *P* < 0.05. Results have been presented as mean ± SD. *P < 0.05; **P < 0.01; ***P < 0.001 in all figures.

## Results

### The characteristics of epithelioid and non-epithelioid hMPM cells

**Fig. 1:**
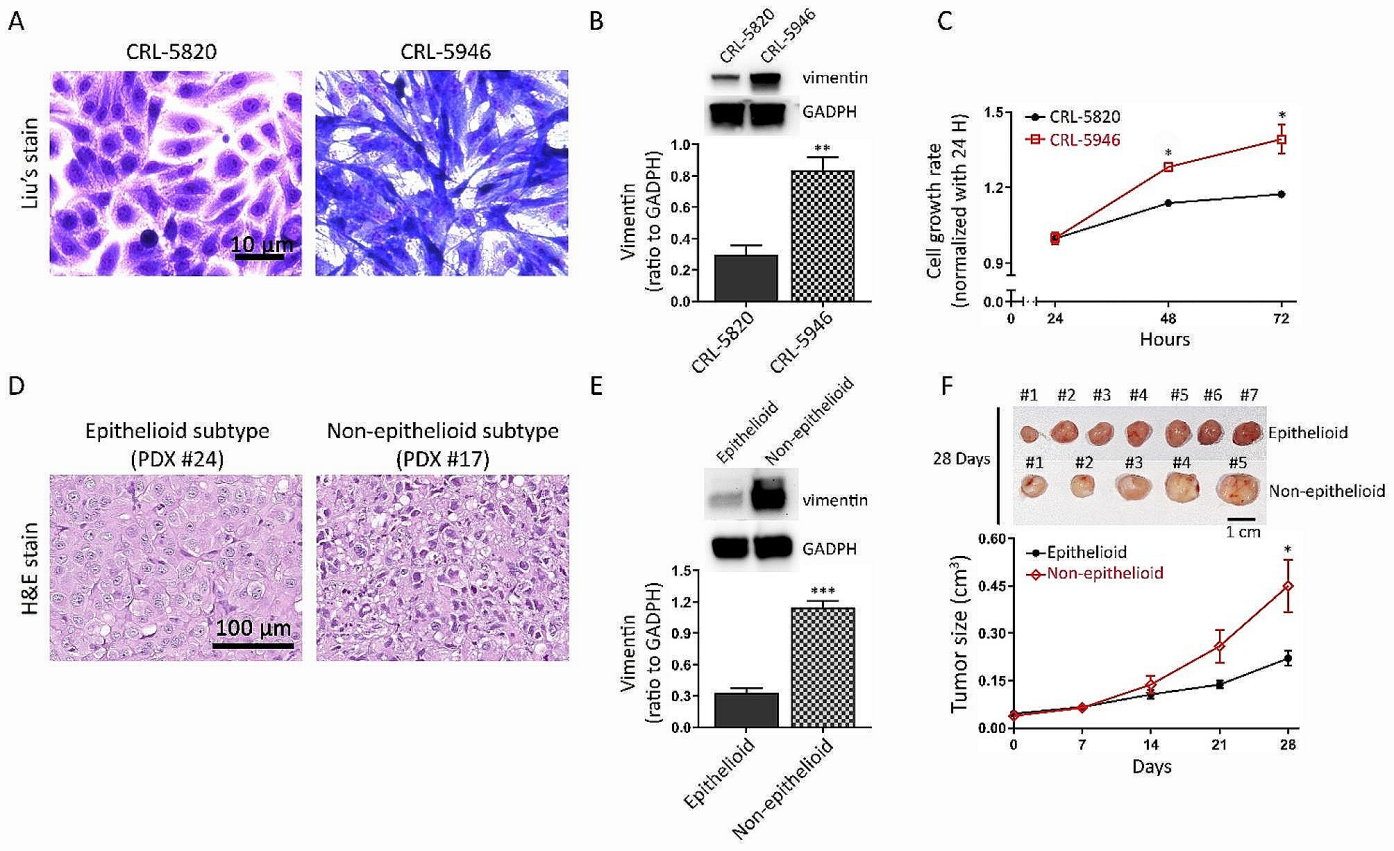
Characteristics of epithelioid and non-epithelioid hMPM cells. The top panel shows the characteristics of the hMPM cell lines (epithelioid subtype, CRL-5820; non-epithelioid subtype, CRL-5946). (**A**) Representative image of Liu’s staining showing the difference in cell morphology between the two hMPM cell lines. (**B**) Comparison of vimentin expression among hMPM cell lines was quantified by immunoblot analysis (*n* = 5). (**C**) 1 × 10^5^ mesothelioma cells were incubated in flat-bottomed culture plates. The proliferation rate was analyzed by counting the number of cells at 24, 48, and 96 h. The bottom panel shows the characteristics of patient-derived xenografts (epithelioid subtype, PDX#24; non-epithelioid subtype, PDX#17) of hMPM. (**D**) Representative images of H&E staining of xenograft tumors from mice. (**E**) Comparison of vimentin expression among hMPM patient-derived xenografts using immunoblot analysis (*n* = 5); (**F**) Implantation of patient-derived xenografts subcutaneously in NOD/SCID mice (*n* = 7). The top figure shows a picture of the tumors 28 d after sacrificing the mice. Tumor size (cm^3^) was measured every 7 days. (* *p* < 0.05, **p < 0.01, *** *p* < 0.005)

This study aimed to investigate the characteristics of epithelioid and non-epithelioid hMPM. Cell morphology was assessed using Liu’s staining of CRL-5820 and CRL-5946 cells, which revealed a cuboid-like (epithelioid) appearance in CRL-5820 cells and a spindle-like (sarcomatoid) appearance in CRL-5946 cells (Fig. 1A). Vimentin, a marker of mesenchymal transition (EMT) (Satelli and Li 2011), showed a significant 2-fold increase in expression in CRL-5946 cells compared to that in CRL-5820 cells (Fig. 1B). Additionally, CRL-5946 cells exhibited a notable increase in proliferation compared with CRL-5820 cells (Fig. 1C). Furthermore, we comprehensively analyzed the distinctive features of human epithelioid and non-epithelioid patient-derived xenografts (PDXs). Our investigation revealed a substantial 4-fold increase in vimentin expression within the non-epithelioid PDX subtype compared to that in the epithelioid PDX subtype (Fig. 1D and E). Moreover, implantation of these PDXs into NOD/SCID mice exclusively demonstrated a significant increase in tumor size in the non-epithelioid PDX group (Fig. 1F).

### The non-epithelioid subtype exhibited resistance to cisplatin treatment

**Fig. 2:**
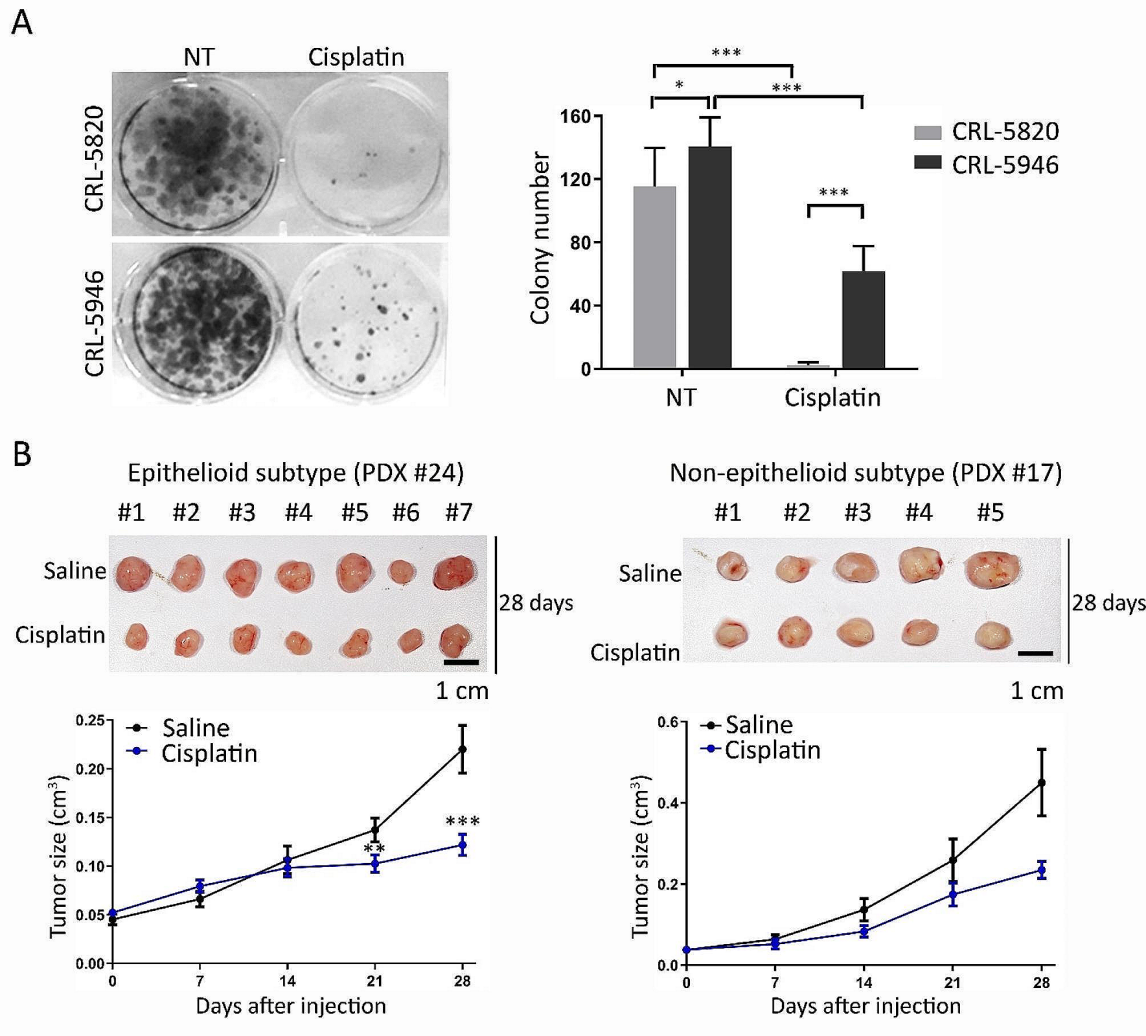
Effects of cisplatin on different histological subtypes of malignant hMPMs. (**A**) Representative images show the comparison of toxicity among different histological subtype. hMPM cells using a colony formation assay. The attached flat bottom 6-well culture plate was seeded with 2 × 10^3^ cells and treated with cisplatin 2 ug/ml for 24 h, then the medium was replaced with a new culture medium. The colonies on the plate were counted 14 days after cisplatin treatment (*n* = 8). (**B**) Comparison of toxicity among different histological subtypes of hMPM patient-derived mouse xenografts. Implanted patient-derived xenografts were subcutaneously injected into NOD/SCID mice, and tumor size (cm^3^) was measured every 7 days (*n* = 5). The upper panel shows a picture of the tumors 28 d after sacrificing the mice. (* *p* < 0.05, **p < 0.01, *** *p* < 0.005)

Patients diagnosed with the non-epithelioid histological subtype of malignant pleural mesothelioma (hMPM) who undergo cisplatin treatment have been associated with inferior overall survival and prognosis-free survival outcomes (Cedres et al. 2021). Given the inherent resistance of the non-epithelioid subtype to cisplatin, we investigated its effects on CRL-5820 and CRL-5946 cells, which represent the epithelioid and non-epithelioid subtypes, respectively. Furthermore, we evaluated the colony formation capacity of these cells following cisplatin treatment. Our findings revealed a significant increase in the number of non-epithelioid CRL-5946 cells compared to epithelioid CRL-5820 cells, highlighting the heightened proliferative capability of non-epithelioid CRL-5946 cells in cisplatin-free or cisplatin-containing conditions, indicating that non-epithelioid CRL-5946 cells are more refractory to cisplatin treatment (Fig. 2A). Furthermore, we implanted epithelioid and non-epithelioid PDXs into NOD/SCID mice and administered a 28-day cisplatin treatment. Regarding PDX tumor implantation, cisplatin significantly inhibited epithelioid tumor growth (*p* < 0.05) and had a trend to reduce the tumor burden in non-epithelioid tumors (Fig. 2B). In clinical practice, stabilization of the tumor burden can be regarded as an important method for disease control, especially in hMPM.

### FK228 was found to inhibit the growth of both epithelial and non-epithelial tumor cells, both in vitro and in vivo

**Fig. 3:**
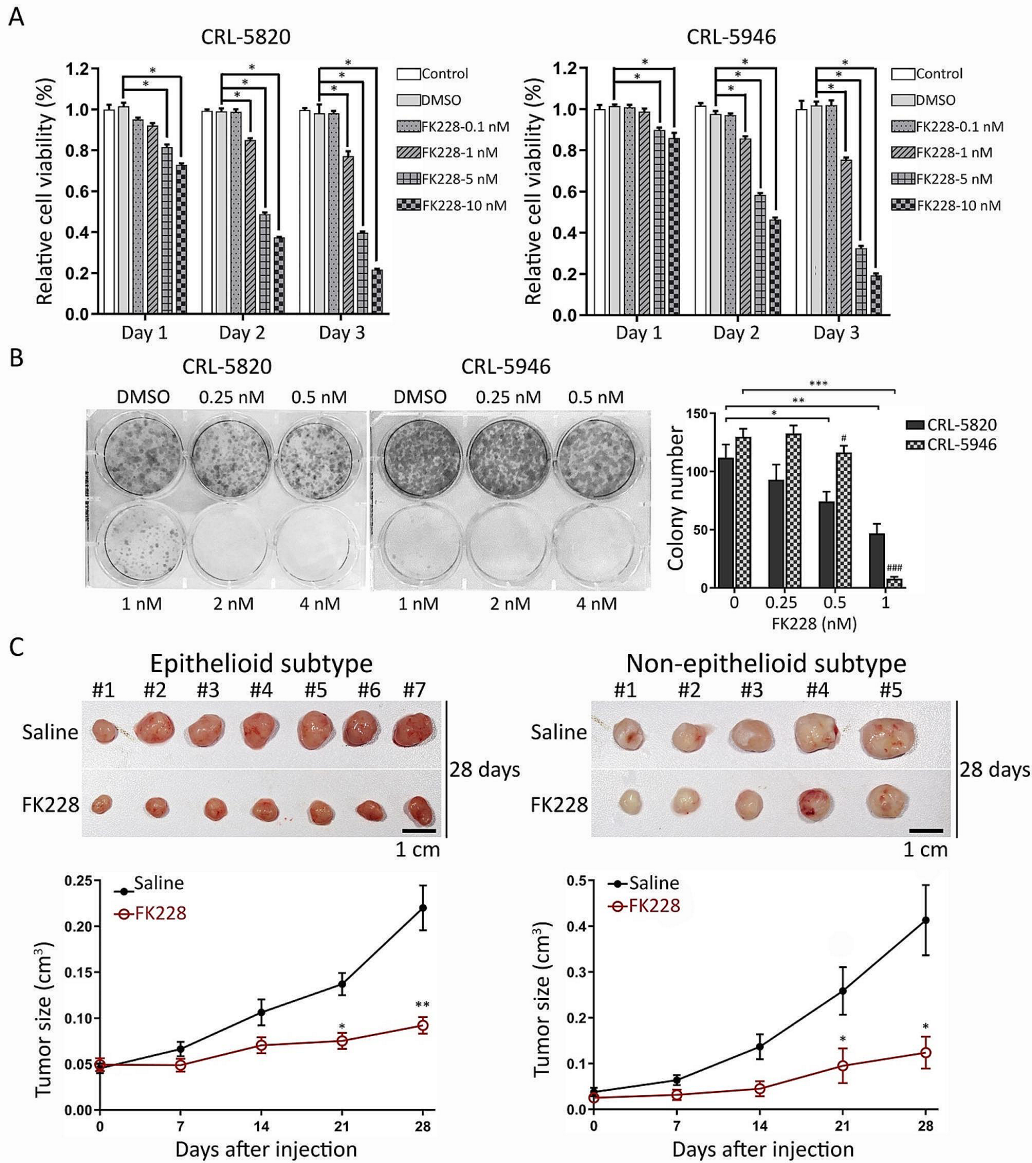
Effects of FK228 on different histological subtypes of malignant hMPMs. (**A**) Short-time toxicity of FK228 on epithelioid (CRL-5820) and non-epithelioid (CRL-5946) hMPM cells at different doses and times (*n* = 4). Relative cell viability was analyzed using the MTT test and presented as the ratio of the treated to the control group. (**B**) Long-term toxicity of FK228 in epithelioid and non-epithelioid hMPM cell lines (*n* = 7). The representative images showing colonies of mesothelioma cells treated with different doses of FK228 after 14 days of incubation by colony formation assay. (**C**) Effect of FK228 on epithelioid (*n* = 7) and non-epithelioid (*n* = 5) subtypes of patient-derived xenografts in mice. Patient-derived xenografts were subcutaneously implanted into NOD/SCID mice. FK228 (2 mg/kg) was injected intraperitoneally on Days 0, 7, 14, and 21. The tumor size was measured every 7 days. The top images show the tumors 28 d after injection and after sacrificing the mice. (* *p* < 0.05, **p < 0.01, *** *p* < 0.005)

To investigate the short-term effects of FK228 on MPM cell proliferation, CRL-5820 and CRL-5946 cells were treated with various concentrations of FK228 (0.1, 1, 5, and 10 nM) for 72 h. Cell viability was assessed using the MTT assay. Our results demonstrated that short-term exposure to FK228 significantly reduced the viability of both CRL-5820 and CRL-5946 cells (Fig. 3A). We also examined the long-term effects of FK228 on colony formation and found that FK228 notably reduced the number of colonies formed by both CRL-5946 and CRL-5820 cells (Fig. 3B). Additionally, in an experiment using epithelioid and non-epithelioid patient-derived xenografts (PDXs) implanted in NOD/SCID mice and treated with FK228 for 28 days, we observed a significant reduction in the average tumor size in both epithelial and non-epithelial implant mice (Fig. 3C). These data show that FK228 effectively inhibits both epithelioid and non-epithelioid mesothelioma cells.

### FK228-induced cell cycle arrest in epithelial and non-epithelial tumor cells

**Fig. 4:**
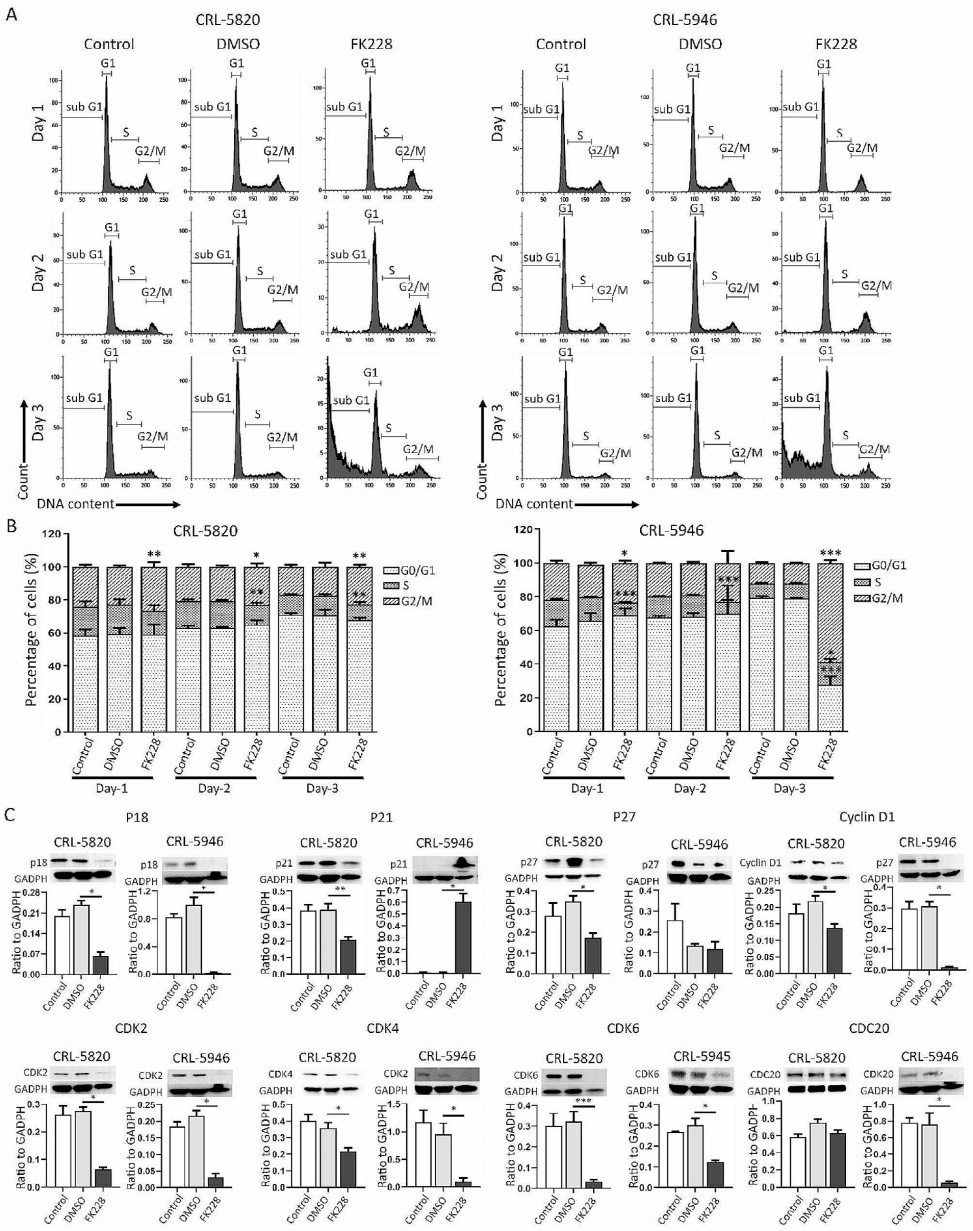
Mechanisms of cell cycle modulation on different histological subtype hMPM cell lines by FK228. Flow cytometry was used for cell cycle analysis. (**A**, **B**) Histograms and bar charts showing a comparison of the distribution of the percentage of cells at different stages of the cell cycle at 1-, 2-, and 3-day time points between control, DMSO, and FK228 (5nM)-treated epithelioid (CRL-5820) and non-epithelioid (CRL-5946) hMPM cells (*n* = 4 for each group). (**C**) Immunoblotting was used to compare the expression levels of p18, p21, p27, cyclin D1, CDK2, CDK4, CDK6, and CDC20 in epithelioid and non-epithelioid mesothelioma cells among control, DMSO, and FK228 (5nM) 3-day-treated mesothelioma cells (*n* = 4 for each group). (* *p* < 0.05, **p < 0.01, *** *p* < 0.005)

Histone deacetylase inhibitors have been shown to inhibit tumor cell growth by inducing cell cycle arrest (Panicker et al. 2010). In this study, we aimed to compare the effects of FK228-induced cell cycle arrest in CRL-5820 and CRL-5946 cells. These cell lines were treated with 5 nM FK228, and the percentage of cells at different stages of the cell cycle was measured on 1, 2, and 3-day time points. Treatment with FK228 resulted in a time-dependent increase in the number of cells in the sub-G1 phase in both CRL-5820 and CRL-5946 cells (Fig. 4A). Following a 3-day treatment with FK228, we observed a significant reduction in the cell population in the S phase in CRL-5820 cells but an increase in CRL-5946 cells. The number of cells in the G_0/1_ phase was also substantially reduced in CRL-5946 cells, as evidenced by the increased expression of P21 in CRL-5946 cells and decreased expression in CRL-5820 cells. We also examined the expression levels of p18, cyclin D1, CDK2, CDK4, and CDK6, and found a significant reduction in all these markers in both cell lines. Furthermore, FK228 induced G_2_/M phase cell cycle arrest in both cell lines, which was more prominent in CRL-5946 cells, as evidenced by the marked decrease in CDC20 expression in CRL-5946 cells, whereas no obvious change was observed in CRL-5820 cells (Fig. 4B and C).

### FK228-induced caspase-dependent cell apoptosis in non-epithelial tumoral cells

**Fig. 5:**
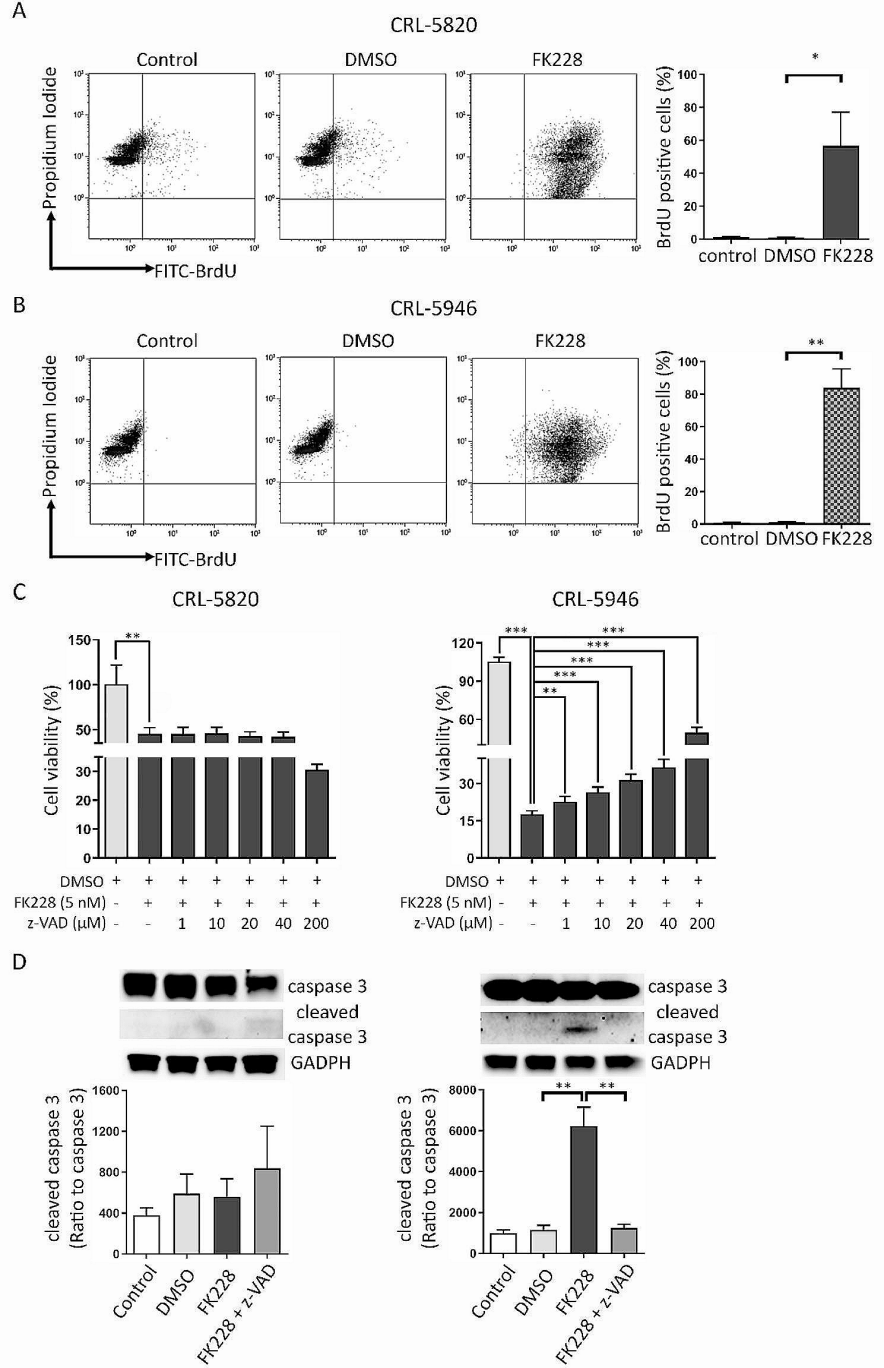
FK228 induced non-epithelioid cell apoptosis by activating a caspase-dependent pathway. hMPM cell lines were treated with FK228 (5 nM) for 72 h. The percentage of apoptotic cells was determined using a BrdU TUNEL assay. (**A**) The number of apoptotic epithelioid cells (CRL5820) was determined using flow cytometry. Data are presented as the mean ± SD (*n* = 4). (**B**) The number of apoptotic non-epithelioid cells (CRL5946) was determined using flow cytometry. Data are presented as the mean ± SD (*n* = 5). (**C**) The MTT assay was performed to measure cell viability. Cell viability (%) was expressed as the percentage of untreated cells or cells treated with FK228. The results were expressed as mean ± SD (*n* = 6). (**D**) Western blot analysis of caspase-3 and cleaved caspase-3. GADPH was used as an internal control (*n* = 6). (_*_p < 0.05, _**_p < 0.01, _***_p < 0.005)

To assess apoptosis, all cells were stained using the BrdU TUNEL assay to detect TdT-mediated BrdU ligation of DNA fragments. FK228 treatment markedly increased the percentage of BrdU + cells in both CRL-5820 and CRL-5946 cell lines after a 3-day treatment (Fig. 5A and B). We examined the role of the apoptotic pathway in FK228-induced cell death using the pan-caspase inhibitor z-VAD-fmk (z-VAD). FK228-induced apoptosis in CRL-5946 cells was partially reversed by treating the cells with z-VAD at concentrations of 1, 10, 20, 40 and 200 µM, but wasn’t in CRL-5820 cells, indicating the involvement of caspase-dependent apoptotic pathways induced by FK228 in CRL-5946 cells (Fig. 5C). Furthermore, we evaluated caspase 3 activation and observed a significant reduction in the formation of cleaved caspase 3 with 200 µM z-VAD treatment (Fig. 5D). FK228 down-regulated the expression of PI3K and Akt, which are involved in the survival pathway, in both epithelioid and non-epithelioid cells (Figure S1).

### FK228 modulated cytokine expression in human mesothelial tumoral cells

**Fig. 6:**
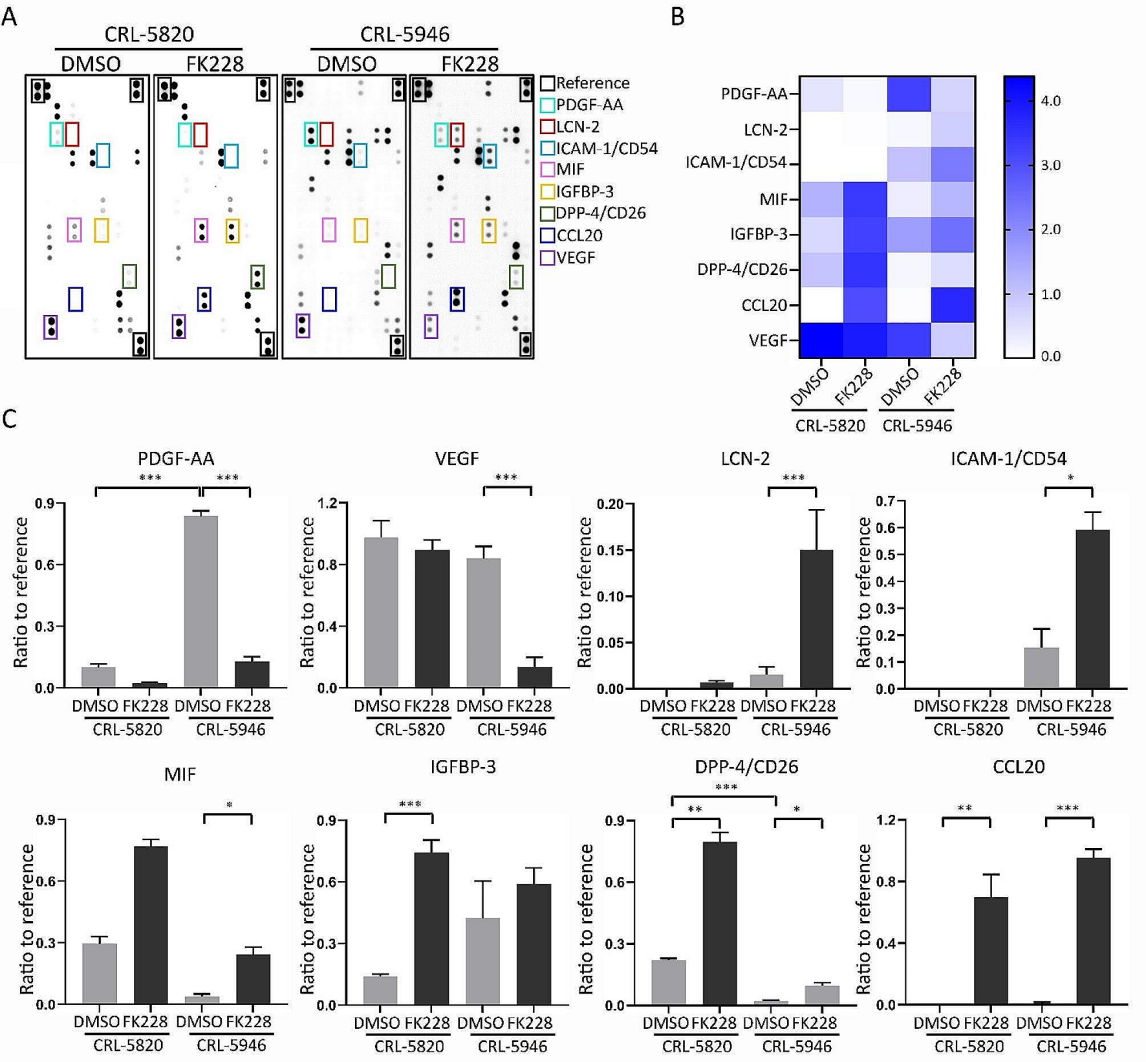
The modulation of cytokine expression on hMPM cell lines induced by FK228. (**A**) Cytokine analysis using human cytokine panels shows the level of cytokine expression on both epithelioid (CRL-5820, *n* = 2, with each protein analyzed twice on a chip) and non-epithelioid (CRL-5946, *n* = 4, with each protein analyzed twice on a chip) hMPM cells treated with DMSO and FK228 (5nM-treated for 3 days). The colored rectangle marked the significantly up-regulated or down-regulated cytokines. (**B**) Heatmap shows the significantly changed cytokines of both subtypes of mesothelioma cells. (**C**) The comparison of significantly regulated cytokines between DMSO and FK228 on both subtypes mesothelioma cells. (_*_p < 0.05, _**_p < 0.01, _***_p < 0.005)

Histone deacetylase inhibitors have recently been recognized not only for their ability to induce tumor cell apoptosis but also for their ability to modify the tumor microenvironment (Li et al. 2021). In this study, we treated two cell lines with 5 µM FK228 for 3 days and collected conditioned medium samples for cytokine analysis using human cytokine panels. The levels of various cytokines released in the conditioned media of CRL-5820 and CRL-5946 cells with and without FK228 treatment were evaluated (Fig. 6A–C). The baseline secretion of PDGF-AA was greater, whereas that of DPP-4/CD26 was lower in CRL-5946 cells. Quantitative data for the cytokines demonstrated significant changes in CRL-5946 cells, including a decrease in vascular endothelial growth factor (VEGF) and platelet-derived growth factor AA (PDGF-AA) and an increase in ICAM1/CD54, LCN2, MIF, DPP4/CD26, and CCL20. In CRL-5820 cells, a significant increase in IGFBP-3, DPP-4/CD26, and CCL20 expression was observed (Fig. 6B and C). These findings indicate that FK228 can alter cytokine secretion in human mesothelial tumor cells, which not only affects the self-growth rate (autocrine effect) but also potentially influences the tumor microenvironment.

## Discussion

The treatment of malignant pleural mesothelioma (MPM) remains challenging, especially for patients with the non-epithelioid subtype, who often experience limited relief with standard therapies (Cho et al. 2014). In this study, we investigated the therapeutic potential of FK228 in human malignant mesothelioma (hMPM). Our findings indicated that FK228 induces apoptotic cell death and inhibits the proliferation of both epithelioid and non-epithelioid hMPM cells via different mechanisms. Furthermore, FK228 significantly inhibited the growth of both epithelioid and non-epithelioid PDXs in immunocompromised NOD/SCID mice. Hence, FK228 has the potential to shed light on hMPM therapy.

Our in vitro morphological analysis confirmed the non-epithelioid subtype of the CRL-5946 cell line and the epithelioid subtype of the CRL-5820 cell line. Consistent with these morphological findings, CRL-5946 cells exhibited increased cell proliferation, EMT activity, and cisplatin resistance compared to CRL-5820 cells. Importantly, FK228 exerted significant short- and long-term cytotoxic effects on both non-epithelioid and epithelioid cells. Our findings from patient-derived xenografts (PDXs) also indicate that non-epithelioid subtype PDXs exhibit higher epithelial-mesenchymal transition (EMT) activity than epithelioid subtype PDXs. Moreover, in vivo implantation of non-epithelioid subtype PDXs in mice resulted in increased cisplatin resistance compared to epithelioid subtype mesothelioma, highlighting the challenges associated with the treatment of this aggressive subtype. To evaluate the efficacy of FK228, we administered repeated injections of the drug to PDX-grafted mice, which effectively inhibited tumor growth in both epithelioid and non-epithelioid PDXs. In both in vitro and in vivo assays, FK228 effectively inhibited both epithelioid and non-epithelioid hMPMs.

In a previous study, we reported significant overexpression of Glucocorticoid-Induced TNFR-Related Protein Ligand (GITRL) and its receptor GITR in non-epithelioid mesothelioma tumors compared to that in epithelioid tumors. Upregulation of GITR/GITRL signaling is associated with enhanced cell proliferation and increased resistance to chemotherapy and radiotherapy, contributing to worse survival in patients with non-epithelioid mesothelioma. Transcriptome analysis revealed that GITR/GITRL signaling is involved in cell cycle regulation and PI3K/Akt activation, promoting tumor cell proliferation (Chan et al. 2021). HDAC inhibitors have been reported to impact cell cycle progression by inhibiting cyclin-dependent kinase (CDK) activation and triggering P21 expression in various studies (Sandor et al. 2000; Richon et al. 2000; Qiu et al. 2000). In our investigation, FK228 treatment modulated the G_0/1_-S transition in CRL-5820 cells and the G_2_/M phase distribution in both cell lines. Inhibition of the G_0/1_-S transition in CRL-5820 cells was associated with the downregulation of the p21 protein. The expression of CDC20 was suppressed in CRL-5946 but not in CRL-5820 cells. Cell cycle analysis demonstrated G_2_/M arrest in both cell lines. Given that the expression of CDC20 may undergo dynamic alterations during different stages of mitotic processing, it might be compatible with the role of CDC20 in regulating spine checkpoint function (Bruno et al. 2022).

The effects of FK228 on cell cycle inhibition in CRL-5946 cells were even more pronounced. Notably, FK228 inhibited the expression of p18, cyclin D1, CDK2, CDK4, and CDK6 in both cell lines. Furthermore, FK228 treatment resulted in a significant increase in p21 expression and a significant decrease in CDC20 expression in CRL-5946 cells, which were not observed in the CRL-5820 cells. These observations highlight the distinct responses of cell cycle regulatory proteins to FK228 treatment in different mesothelioma cell lines. Carcinogenesis involves both genetic alterations and epigenetic modifications, rendering the mechanisms of the anticancer effects of HDAC inhibitors diverse and dependent on the cancer type (Eckschlager et al. 2017). Further research is needed to elucidate the specific mechanisms underlying the actions of HDAC inhibitors, particularly in the context of different cancer types, and to develop more targeted and effective therapeutic strategies.

The PI3K/Akt pathway plays a crucial role in regulating cell survival and proliferation and is commonly dysregulated in various human cancers, making it an attractive therapeutic target (Liu et al. 2009). Our transcriptome analysis revealed upregulation of the PI3K/Akt pathway in non-epithelioid mesothelioma cells, suggesting its potential association with increased cell proliferation and resistance to therapy (Chan et al. 2021). Notably, FK228 and its analogs have been discovered to have dual functions as histone deacetylases and PI3K inhibitors (Saijo et al. 2012). Our study demonstrated that FK228 induced apoptosis in human mesothelioma cells, accompanied by a significant decrease in PI3K and Akt protein expressions after treatment, indicating the inhibition of this pathway by the drug. Interestingly, we further investigated caspase-dependent and -independent cell apoptosis using the pan-caspase inhibitor z-VAD-FMK. Our findings revealed that FK228 triggered caspase-independent apoptosis in non-epithelioid cells but not in epithelioid cells. This indicates potential differences in the mechanism of cell apoptosis induction by FK228 between the two mesothelioma cell types, highlighting the complexity of FK228’s effects on cell death pathways.

The tumor microenvironment, which is composed of tumor cells, immune cells, stromal cells, and surrounding tissues, plays a crucial role in cancer progression rather than being a mere bystander (Anderson and Simon 2020). For instance, PDGF-AA has been shown to enhance tumor progression in osteosarcoma by interacting with the PDGF-α receptor (Sulzbacher et al. 2003). Human cytokine panel analysis revealed that PDGF-AA levels were significantly higher in non-epithelioid CRL-5946 cells than in epithelioid CRL-5820 cells. This finding provides valuable evidence explaining the aggressive cell growth observed in non-epithelioid cells, suggesting that elevated PDGF-AA levels in the tumor microenvironment of non-epithelioid mesothelioma cells may contribute to enhanced tumor progression and aggressive behavior. Additionally, vascular endothelial growth factor (VEGF) is a critical angiogenic factor in tumor initiation and progression (Yang and Cao 2022). In our study, VEGF secretion was significantly inhibited in non-epithelioid CRL-5946 cells following FK228 treatment, suggesting that FK228 may have anti-angiogenic effects that potentially hinder tumor growth and metastasis. Furthermore, IGFBP-3 and its receptor have been reported to have anti-cancer and anti-metastatic effects, attenuating tumor progression (Cai et al. 2020). *Wang et al.*. provided evidence that overexpression of IGFBP-3 can induce cell apoptosis and enhance the cisplatin response in lung cancer (Wang et al. 2017). In this study, we observed a significant increase in IGFBP-3 levels in epithelioid CRL-5820 cells following FK228 treatment. However, the elevation of IGFBP-3 secretion was not altered in non-epithelioid CRL-5946 cells, implying potential differences in the FK228 response between the two mesothelioma cell types. We also observed paradoxical results in that FK228 treatment induced the secretion of dipeptidyl peptidase 4 (DPP4)/CD26, Lipocalin 2 (LCN-2), and chemokine (C-C motif) ligand 20 (CCL20) in mesothelial cells. These markers are overexpressed in several cancer types and are associated with cancer development (Kadomoto et al. 2020; Enz et al. 2019; Hu et al. 2018). However, further research in the detailed mechanisms underlying FK228-mediated changes in the tumor microenvironment is required.

## Conclusion

In conclusion, our study highlights the potential therapeutic effects of FK228 on hMPM, including both epithelioid and non-epithelioid subtypes. The distinct responses to FK228 treatment observed in different subtypes of mesothelioma cell lines underscore the importance of further research to elucidate the underlying mechanisms and develop more targeted and effective therapeutic strategies for this aggressive disease. Our findings provide valuable insights into the complex interplay between the tumor microenvironment and FK228 treatment, emphasizing the need for tailored approaches to improve outcomes for patients with hMPM. Further investigations are warranted to translate these findings into clinically relevant therapeutic options and to advance precision medicine for the treatment of mesothelioma.

## Data Availability

The authors confirm that the data supporting the findings of this study are available within the article and its supplementary materials.

## Abbreviations

hMPM: Human malignant pleural mesothelioma
PDX: Patient derived xenograft
